# Orange juice containing *Pediococcus acidilactici* CE51 modulates the intestinal microbiota and reduces induced inflammation in a murine model of colitis

**DOI:** 10.1038/s41598-023-45819-4

**Published:** 2023-10-28

**Authors:** Karolinny Cristiny de Oliveira Vieira, Ana Beatriz Batista da Silva, Suelen Aparecida Felício, Fábio Santos Lira, Caíque de Figueiredo, Eugenia Bezirtzoglou, Valéria Cataneli Pereira, Wilson Romero Nakagaki, Gisele Alborghetti Nai, Lizziane Kretli Winkelströter

**Affiliations:** 1 Health Sciences Faculty, UNOESTE (University of Western Sao Paulo), 700, Jose Bongiovani St., Cidade Universitária, Presidente Prudente, Sao Paulo 19050-920 Brazil; 2 Master in Health Science, UNOESTE (University of Western Sao Paulo), 700, Jose Bongiovani St., Presidente Prudente, Sao Paulo 19050-920 Brazil; 3 Department of Physical Education, Faculdade de Ciências e Tecnologia, Universidade Estadual Paulista, UNESP, Rua Roberto Simonsen, 305, Presidente Prudente, Sao Paulo 19060-900 Brazil; 4 Laboratory of Hygiene and Environmental Protection, Department of Medicine, Democritus University of Thrace, Dragana, 68100 Alexandroupolis, Greece

**Keywords:** Microbiology, Gastroenterology

## Abstract

The management of inflammatory bowel diseases has been widely investigated, especially ulcerative colitis. Thus, studies with the application of new probiotic products are needed in the prevention/treatment of these clinical conditions. The objective of this work was to evaluate the effects of probiotic orange juice containing *Pediococcus acidilactici* CE51 in a murine model of colitis. 45 male Swiss lineage mice were used, divided into five groups (n = 9): control, colitis, colitis + probiotic (probiotic orange juice containing CE51), colitis + placebo (orange juice) and colitis + sulfasalazine (10 mg/kg/Weight). The induction of colitis was performed with dextran sodium sulfate (3%). The treatment time was 5 and 15 days after induction. Histopathological analysis, serum measurements of TNF-α and C-reactive protein and metagenomic analysis of feces were performed after euthanasia. Probiotic treatment reduced inflammation in the small intestine, large intestine and spleen. The probiotic did not alter the serum dosages of TNF-α and C-reactive protein. Their use maintained the quantitative ratio of the phylum *Firmicutes/Bacteroidetes* and increased *Lactobacillus helveticus* with 15 days of treatment (p < 0.05). The probiotic orange juice containing *P. acidilactici* CE51 positively modulated the gut microbiota composition and attenuated the inflammation induced in colitis.

## Introduction

Inflammatory bowel diseases (IBD) are described as chronic conditions, marked by recurrent episodes of gastrointestinal tract inflammation, which can be included ulcerative colitis (UC) that takes an incidence rates that vary 2.2 to 19.2 per 100,000 inhabitants in the world^1,2^.

Ulcerative colitis, ulcerous colitis or ulcerative rectum colitis, is an inflammation limited to the mucosa, located most often from the rectum to the proximal colon. The main cause of ulcerative colitis is believed to be continuous inflammation in the mucosa, which is associated with significant nutritional disorders, leading to ulcerations and bleeding^1,3^. The current treatment of UC is mainly based on the use of anti-inflammatory drugs, immunomodulators, nutritional supplements and surgery^4^.

Sulfasalazine is the widely used drug for remission of ulcerative colitis. This medicine is composed by the combination of 5-aminosalicylic acid (5-ASA) with the sulphapyridine molecule, which transports the active ingredient to the colon and, in it, exerts anti-inflammatory action. In addition to, the sulphapyridine has also antibacterial action. It is possible that sulfasalazine attenuates ulcerative colitis inflammation by eliminating free radicals, and thus inhibiting the production of prostaglandins and leukotrienes and / or reducing neutrophil chemotaxis and superoxide production^5^.

Ulcerative colitis is also associated with a decrease in *Lactobacillus* spp. and *Bifidobacterium* spp., which suggests the need for research to assess the use of functional food substances that contains biologically active substances with clinical health benefits^6,7^.

Among the functional foods, there are probiotics, used mainly in dairy products, which may be associated with reducing the risk of chronic degenerative and non-communicable diseases^8^.

As defined by the World Health Organization (WHO)/Food and Agriculture Organization (FAO)^9^, probiotics are considered living microorganisms which, when administered in adequate amounts, confer benefits on the health of the host. Probiotics have the potential to maintain intestinal balance, preserve immune health and act with benefits in the treatment and prevention of inflammatory bowel diseases, especially for ulcerative colitis^8,10^.

The demand for probiotic non-dairy foods and drinks has grown due to the greater emergence of individuals with allergies to milk protein and lactose intolerance. Due to the scarcity of these products, the food industry has sought to innovate with probiotic products derived from vegetables. With that orange juice has become a great option due to the ease of obtaining it, as it is the most consumed juice in the world^11^.

In the pharmaceutical industry, there are formulations that comprise *Pediococcus acidilactici* as a probiotic to reduce symptoms of adverse gastrointestinal conditions^12^. Its effectiveness lies in the anti-inflammatory and anti-oxidative action against the stress caused in the intestinal mucosa, which makes *Pediococcus* spp. species as convenient methods in the prevention of intestinal disorders, such as ulcerative colitis^13–15^.

Given the current relevance of using *Pediococcus* spp. for treating and preventing IBD, it presents an intriguing therapeutic possibility for ulcerative colitis. It is still necessary to develop more consistent in vivo research through randomized controlled studies addressing, together, the main clinical, microbiological and immunological parameters to ensure the use of new *Pediococcus acidilactici* strains as useful tools in the promotion of human health^16^.

In this context, the preponderance of evidence suggests that marked alterations in diet can perturb the taxonomical configuration of the microbiota within a few days^17^. According to David et al.^18^, short-term changes (4 days) on the human diets was sufficient to introduce distinct community-wide alterations of the intestinal microbiota.

Although few studies have been published on the applicability of *Pediococcus acidilactici* as a probiotic strain related to UC, none of them evaluated the effects of probiotic orange juice containing a new isolate on the animal model of ulcerative colitis, named as *Pediococcus acidilactici* CE51. Thus, the objective of this study was to evaluate the anti-inflammatory response and gut modulation of probiotic orange juice treatment in a short-term consumption (5 days) and (15 days) in gut inflammation induced in murine model.

## Material and methods

### Elaboration of probiotic drink

The probiotic drink was prepared according to Vieira and collaborators^11^. Concentrated orange juice (Campo Largo, Brazil) without added preservatives and sugar was used, and inoculated *P. acidilactici* CE51 at 10^8^ CFU/mL^11^. The juice was stored in a refrigerator at 5 °C (Gelopar, Brazil) for up to 07 days for use in animal experimentation.

### Animals and experimental groups

Male *Mus musculus* (swiss) eight-week-old mice weighing 34.5 to 54.0 g, from the animal house of the University of Western Sao Paulo were used. The animals were kept in polypropylene boxes, on a shelf with an air filtration system and temperature and relative humidity control (23 °C; 60–70% humidity), light (12 h light/dark cycle), fed with mice feed (NUVILAB CR-1, QUIMTIA, Brazil) and filtered drinking water ad libitum^19,20^ and acclimated to the conditions for at least seven days before experimental manipulation. The experiments took place during the clear phase of the cycle and were carried out in accordance with the approval of the Ethics Committee on the Use of Animals of University of Western Sao Paulo (CEUA/Unoeste—nº 5897). This study was carried out in compliance with the ARRIVE guidelines. A total of 45 mice were used, randomly distributed into five groups containing nine animals, divided as shown in Table 1.

**Table 1:** Description of the experimental groups.

Experimental groups	Description
Control (C)	Healthy animals that did not receive the products under study
Colitis (CL)	Animals with chemically induced colitis that have not received any treatment
Colitis + probiotic (CLPR)	Animals with chemically induced colitis and which received orange juice containing probiotic *Pediococcus acidilactici* CE51
Colitis + placebo (CLP)	Animals with chemically induced colitis that received only orange juice (placebo)
Colitis + sulfasalazine (CLS)	Animals with chemically induced colitis that received the sulfasalazine drug

A total of 45 animals was divided into 5 experimental groups, namely, Control (C): Did not receive induction of colitis by DSS and no treatment; Colitis (CL): Induction of colitis by DSS and absence of treatment; Colitis + Probiotic (CLPR): Induction of colitis by DSS and treatment with *P. acidilactici* CE51; Colitis + Placebo (CLP): Induction of colitis by DSS and treatment with only orange juice; Colitis + Sulfasalazine (CLS): Induction of colitis by DSS and treatment with sulfasalazine drug.

### Experimental colitis induced by dextran sodium sulfate

**Figure 1:**
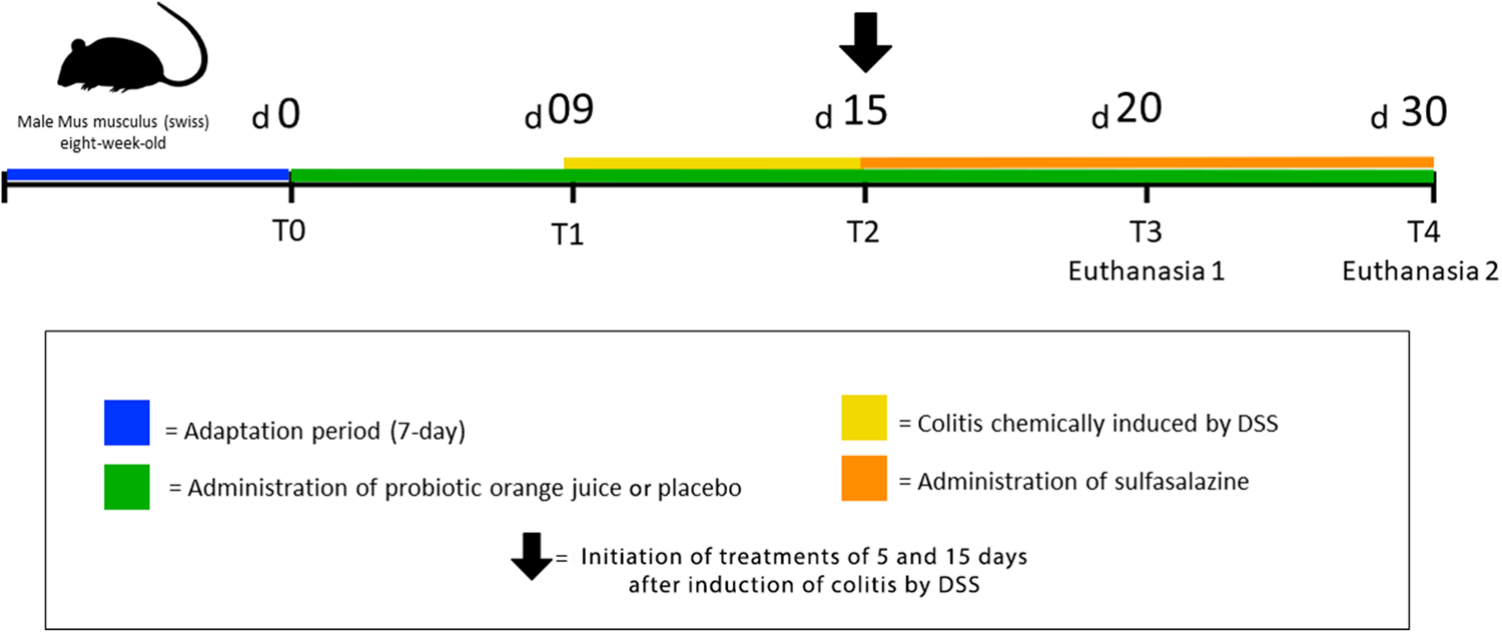
Experimental design. The experiment was carried out with 45 animals divided in 5 experimental groups Control (C); Colitis (CL); Colitis + Probiotic (CLPR; Colitis + Placebo (CLP); Colitis + Sulfasalazine (CLS) for 37 days divided in the following stages: Adaptation period of 7 days (Blue line); T0/d0: Initiation of administration of probiotic orange juice containing *P. acidilactici* CE51 for the CLPR group, or orange juice (placebo) for the CLP group, which lasted until the last day of the experiment (Green line); T1/d09: Beginning of the period of induction of ulcerative colitis by DSS, which lasted 6 consecutive days (Yellow line); T2/d15: The arrow indicates the withdrawal of the DSS and the beginning of the treatments that lasted 5 and 15 days. In this period also started treatment with sulfasalazine (Orange line); T3/d20: Exact time when the first euthanasia occurred (Euthanasia 1) after treatment with probiotic orange juice, placebo or sulfasalazine for 5 days; T4/d30: Exact time when the second euthanasia (Euthanasia 2) occurred after treatment with probiotic orange juice, placebo or sulfasalazine for 15 days. *d* Day (Above the colored lines); *T* Time (Below the colored lines); *Black arrow* Induction of ulcerative colitis by DSS.

The experiment period was divided into four phases as shown in Fig. 1: (1) adaptation (7 days), (2) treatment with probiotics or placebo (9 days), (3) induction of colitis (CL) and treatment with probiotic (CLPR), placebo (CLP) or sulfasalazine (CLS) (6 days) and 4) treatment with probiotic, placebo or sulfasalazine (5 and 15 days).

The experimental colitis was chemically induced, through the administration of dextran sodium sulfate (DSS) (Sigma-Aldrich, St. Louis, MO, USA). The DSS (3%) was dissolved (15 g) in the filtered water (500 mL) supplied daily to the animals, and administered over a period of 6 days^21,22^. The animals were monitored daily for weight and clinical signs.

The severity of colitis was measured every day taking into account the three parameters and their respective scores of the disease activity index (DAI). The weight of the animals was evaluated by an analytical balance (Gehaka, BT8000, Brazil), classified by the scores: body weight loss (score 0, no weight loss compared to initial weight; (1) weight loss within 1–5%; (2) weight loss within 5–10%; (3) weight loss within 10–20%; (4) greater than 20% weight loss). The stool consistency by observation of the feces in the cages (score 0, normal—solid consistency); 2, loose stool but with some solidity; 4, diarrhea). For the analysis of occult blood in the feces, the Meyer Method was used in order to identify the presence of hemoglobin in the sample^23^. The presence of occult/visible blood in the stools was also classified by the following score: score 0, negative; 2, fecal occult blood test positive; 4, gross bleeding^24,25^.

The animals in the probiotic (CLPR) and placebo (CLP) groups received 50 µL of the products under study daily, orally by means of a micropipette (Single channel Pipette, LABMATE Pro, Poland) (enough to guarantee a daily intake of 10^8^ CFU of the probiotic microorganisms in probiotic orange juice group). The animals of the Sulfasalazine group (CLS) received the drug (Sigma-Aldrich, St. Louis, MO, USA) daily, orally through a micropipette (Single channel Pipette, LABMATE Pro, Poland) (10 mg/kg/weight). The Control (C) and Colitis (CL) groups did not receive any of the treatments, only water and food. After 5 days of treatment, four animals from each group were euthanized, and with 15 days, the remaining five animals from each group. For euthanasia, anesthetics Ketamine Hydrochloride (10%) (Rompun, Bayer HealthCare, Kansas, MO, USA and Xylazine Hydrochloride (2%) (Bayer, Leverkusen, Germany) were administered intraperitoneally (75 e 10 mg/kg). Laparotomy was performed and the organs of the gastrointestinal system (the small and large intestines, stomach, liver and spleen) were removed and opened longitudinally for later macroscopic and histological analysis.

### Histological analysis

After each treatment period (5 and 15 days), the animals were euthanized. The small and large intestines were removed and then, the lengths were measured. The stomach, liver and spleen were also collected to evaluate the potential general effect of the induced inflammation in other parts of the body and possible toxic effect of probiotic juice.

All organs were cleaned (fats and tissues adhered to the wall were removed), washed in running water to remove feces, fixed by immersion in a solution of 10% buffered formalin for 48 h, processed routinely and embedded in paraffin. Histological sections of 5 μm thick were stained with Hematoxylin–eosin (HE) for analysis under light microscopy (NIKON Labophot, Japan) by one blind trained researcher to the treatment.

These general parameters were evaluated in the stomach, and small and large intestines with their respective scores were interstitial inflammatory infiltrate (0 = absent, 1 = mild, 2 = moderate, 3 = intense) and type of inflammatory cell present (polymorphonuclear and/or mononuclear); tissue congestion (0 = absent, 1 = mild, 2 = moderate, 3 = severe). The following parameters were also specifically analyzed: Intestine (small and large) the following parameters were also evaluated: lymphoid hyperplasia (0 = absent; 1 = present); epithelial ulceration (0 = absent; 1 = present and small; 2 = present and extensive); regenerative atypias of the epithelium (0 = absent; 1 = present); and intestinal wall necrosis (0 = absent; 1 = present); Spleen: white pulp hyperplasia (0 = absent; 1 = present) and red pulp hyperplasia (0 = absent; 1 = present); Liver: steatosis (0 = absent; 1 = present) and its pattern: macrovesicular; microvesicular; or mixed (macro and microvesicular)^26^.

Histomorphometry analysis was performed, obtaining 3 selected sections of each animal. The height (μm) of 10 villi and 10 crypts of each animal was obtained by measuring the vertical distance from the upper extremity to the lower limit of each. The villus and crypt height values for each animal were represented by the mean values of the three histological sections^27^.

The images of the histological sections were captured in a 100 × magnification by a digital camera connected to a light microscope (LEICA DM750, Leica Microsystems, Germany). The digital images were analyzed using appropriate software (Image J® 1.50b, National Institutes of Health, USA) by one blind trained researcher^27^.

### Serum dosage for TNF-α and C-reactive protein

The animals' blood was collected by cardiac puncture under anesthesia, and then centrifuged (15 min at 1000×*g* at room temperature) (FANEM®, 206 BL, Brazil). The serum was collected and stored at – 20 °C until analysis. The concentration of TNF-α and C-reactive protein (CRP) were determined by the enzyme-linked immunosorbent assay (ELISA) with commercial kits (FineTest, Wuhan, China and Millipore, Billerica, MA, USA, respectively) with intra- and inter-assay variations (CV%) of < 8% and < 10% to TNF-α and ± 8% and ± 7% to CRP. Briefly, the serum samples were incubated with antibodies (anti-TNF or anti-PCR) and labelled with horseradish peroxidase for subsequent enzymatic reaction with tetramethylbenzidine. The reaction was stopped by the addition of acidic solution and the resulting absorbance was measured spectrophotometrically at 450 nm (SpectraMax® PLUS 384 Microplate Reader, MOLECULAR DEVICES, EUA).

### Metagenomic analysis of the intestinal microbiota

A stool pool of the middle intestine of 4 (treatment = 5 days) and 5 (treatment = 15 days) mice per treatment (Control, Colitis, Probiotic, Placebo and Sulfasalazine) were sampled. The intestinal content (feces) of each animal was collected, stored individually in microtubes free of DNases and RNases and, immediately, frozen at – 20 °C (Freezer Indrel Scientific, Brazil) for subsequent metagenomic analysis. DNA extraction and DNA sequencing analysis of the samples was performed by Neoprospecta Microbiome Technologies (Florianópolis, SC, Brazil) using a molecular technique based on the sequencing of the 16S rRNA gene, amplifying the V3-V4 regions using 341F/806R primers, through the Illumina MiSeq sequencer. Sequence of primers were 341F 5′-CCTAYGGGRBGCASCAG-3′ and 806R 5′-GGACTACNNGGTATCTAAT-3′. The DNA concentration of the amplicon pool was estimated with Picogreen dsDNA assays (Invitrogen, USA), and then the pooled libraries were diluted for accurate quantification of qPCR using KAPA Library Quantification Kit for Illumina platforms (KAPA Biosystems, Woburn, MA, USA).

The library bank was adjusted to a final concentration of 11 pM and sequenced in a MiSeq system, using the Illumina primers provided in the kit. A 300 nt single-ended run was performed using a V2 × 300 sequencing kit.

Sequence data were processed and analyzed with QIIME [Quantitative Insights Into Microbial Ecology, version 2022.2.0 (https://qiime2.org/)]. On average, a total of 153,269 raw reads were sequenced. Initially, during the demultiplexing and trimming steps, low quality reads were removed, such as reads up to Q30, unsatisfactory length reads, and chimeras were removed with QIIME. After this process, the dataset contained an average of 106.918 raw reads. The clean readings were used in defining the Amplicon Sequence Variant (ASV).

To measure the rates present in the samples, a predictor model of the V3 and V4 region was used. Heatmaps and barplots of relative abundance of ASVs were generated with Python (version 3.7) through codes developed by the company ByMyCell Inova Simples Ltda.

### Statistical analysis

All analyzed data were submitted to the normality test using the Kolmogorov–Smirnov test. The animal sample size calculation was performed according to Arifin and Zahiruddin^28^.

The comparison between two groups was performed using the Student's t test for unpaired data or the Mann–Whitney test. When three or more groups were compared, analysis of variance (ANOVA) was used, followed by the Newman-keuls post-test to verify the difference between the groups. The results were considered significant for p < 0.05. The statistical program used was GraphPad Prism 3.0.

### Ethics approval

The experiments were carried out in accordance with the approval of the Ethics Committee on the Use of Animals of University of Western Sao Paulo (CEUA / Unoeste—nº 5897). This study was carried out in compliance with the ARRIVE guidelines.

## Results

### Beneficial effect of probiotic orange juice on DAI analysis in DSS-induced colitis

**Figure 2:**
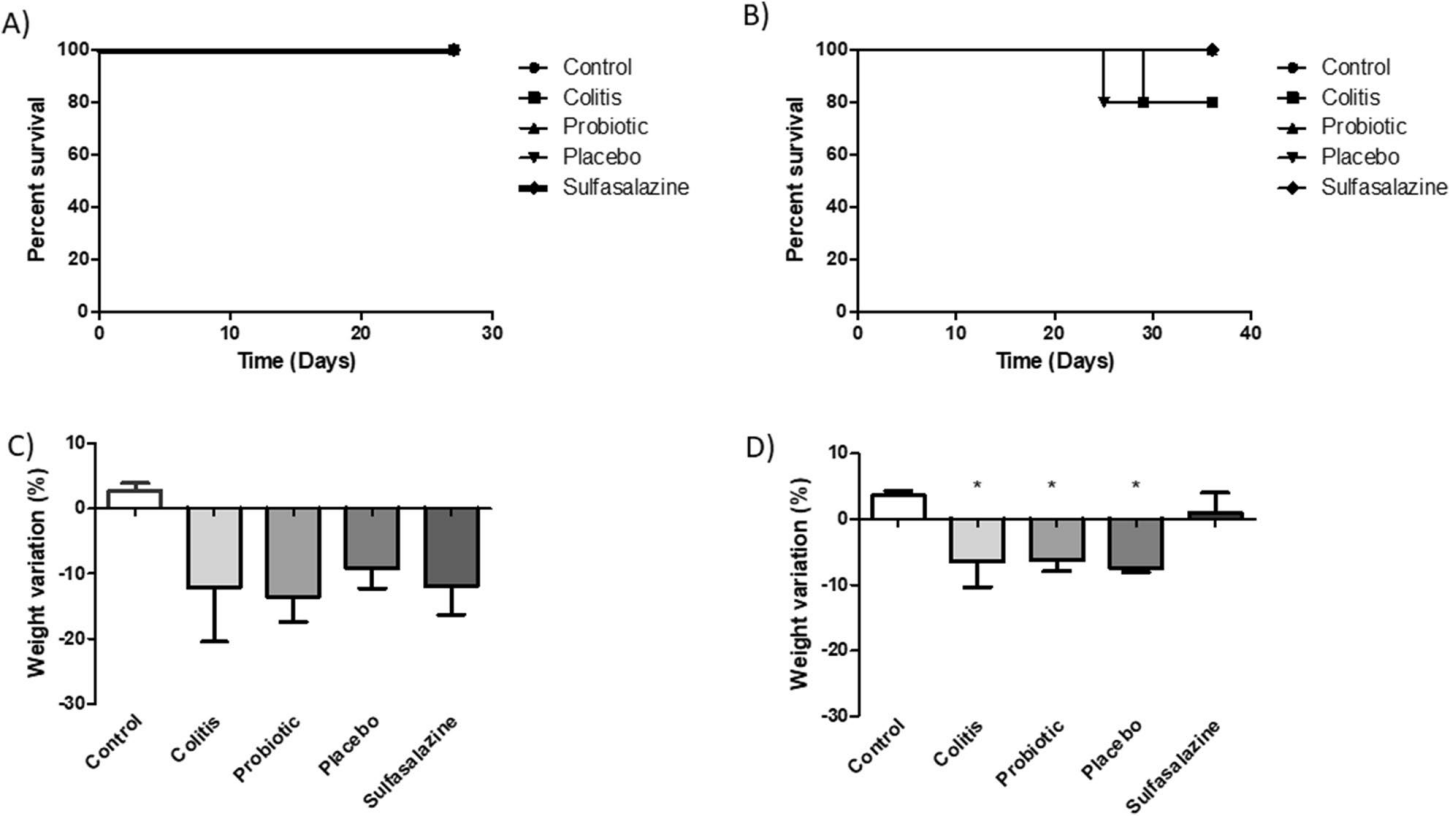
Beneficial effect of probiotic orange juice on DAI analysis in DSS-induced colitis. Survival curve (**A**, **B**) and weight variation (**C**, **D**) of mice treated with 5 days (left side) (n = 4) and 15 days (right side) (n = 5) with probiotic (orange juice containing *Pediococcus acidilactici* CE51), placebo (orange juice) or sulfasalazine (10 mg/kg/weight) after DSS administration (3%) for induced colitis. The control group was not submitted to the colitis protocol. Comparison was done between all groups of each treatment period. *Statistically significant difference in weight variation between control group and groups: colitis, placebo and probiotic.

Clinical signs, symptomatologic parameters and macroscopic damage were analyzed according to the disease activity index criteria. There was a change in stool consistency and the presence of visible and occult blood was observed for up to 5 days after induction of colitis with DSS. However, the parameters were more intense in the colitis, placebo and sulfasalazine groups. Despite the loss of two mice (1 colitis group and 1 placebo group), no significant difference was observed in the survival curve between the groups after 5 and 15 days of treatment (Fig. 2A,B).

After 5 days of treatment, no significant changes were observed in the weight variation of experimental groups of animals during the period before induction of colitis and after treatment with probiotic, placebo and sulfasalazine (Fig. 2C). However, the colitis, probiotic, placebo groups showed a significant decrease in weight in comparison to control group after 15 days of treatment (Fig. 2D).

### Attenuation of inflammation by probiotic orange juice treatment in histopathological analysis in DSS-induced colitis with 5 days of treatment

**Figure 3:**
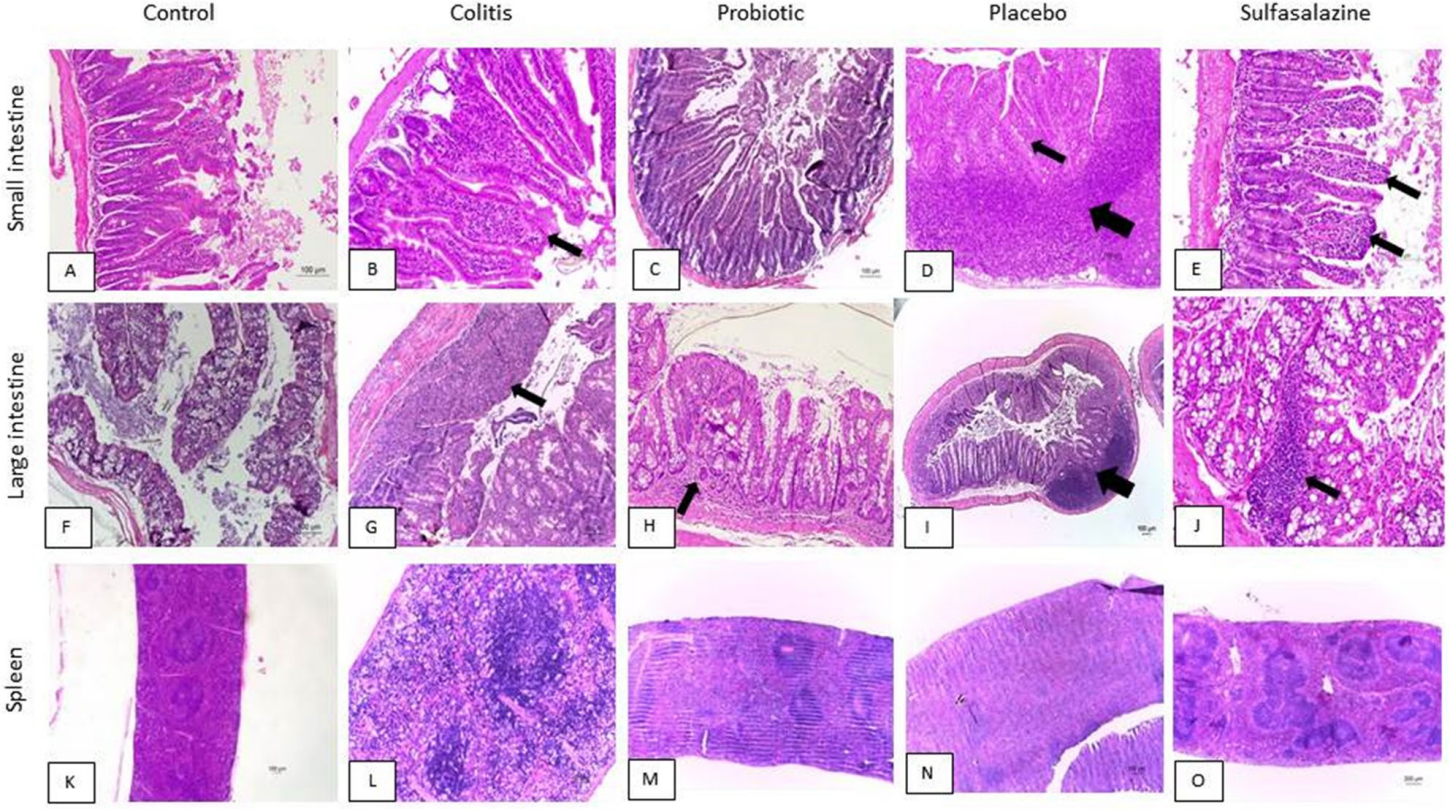
Attenuation of inflammation by probiotic orange juice treatment in histopathological analysis in DSS-induced colitis with 5 days of treatment. Photo microscopy of the small intestine: (**A**) Normal mucosa (group C animal). (**B**) Moderate inflammation (arrow) (animal in the CL group). (**C**) Normal mucosa (animal in the CLPR group). (**D**) Lymphoid hyperplasia (wide arrow), and moderate inflammation (thin arrow) (animal in the CLP group). (**E**) Mild inflammation (arrows) (animal in the CLS group). Hematoxylin–eosin, 100 × magnification. Large intestine: (**F**) Normal mucosa (group C animal) (Hematoxylin–eosin, 100 × magnification). (**G**) Ulcerated area of the mucosa (arrow) and transmural inflammation (animal in the CL group) (Hematoxylin–eosin, 100 × magnification). (**H**) Mild inflammation of the mucosa (arrow) (animal in the CLPR group) (Hematoxylin–eosin, 100 × magnification). Spleen: (**I**) Lymphoid hyperplasia (arrow) (animal in the CLP group) (Hematoxylin–eosin, 40 × magnification). (**J**) Mild inflammation of the mucosa (arrow) (animal in the CLS group) (Hematoxylin–eosin, 100 × magnification). (**K**) Normal splenic parenchyma (group C animal) (Hematoxylin–eosin, 40 × magnification). (**L**) Exuberant white pulp hyperplasia with a “starry sky” aspect (animal in the CL group) (Hematoxylin–eosin, 100 × magnification). (**M**) White pulp hyperplasia (animal in the CLPR group) (Hematoxylin–eosin, 40 × magnification). (**N**) White pulp hyperplasia (CLP group animal) (Hematoxylin–eosin, 40 × magnification). (**O**) White pulp hyperplasia (CLS group animal) (Hematoxylin–eosin, 40 × magnification).

Histopathological analyzes were performed on the stomach, small intestine, large intestine, liver and spleen (Table 2 and Fig. 3). No significant changes were found for stomach and liver (data not shown).

**Table 2:** Histopathological analysis.

Treatment time	Groups	Small intestine		Large intestine		Spleen	
		Inflammation n/N (%)	Villus measures (μm)	Crypt measures (μm)	Inflammation n/N (%)	Presence of ulceration n/N (%)	White pulp hyperplasia n/N (%)
5 days	Control	0/4 (0)^b,c,d,e^	736,00 ± 161^c,d^	206,08 ± 34^d^	0/4 (0)^b,d,e^	0/4 (0)^b,d,e^	1/4 (25)^b^
	Colitis	3/4 (75)^a,c,d,e,^	782,51 ± 76^c,d^	370,31 ± 86^d^	1/4 (25)^a,c,d,e^	1/4 (25) ^a,c,d,e^	3/4 (75)^a,c,d,e^
	Probiotic	1/4 (25)^a,b,d^	975,88 ± 74^a,b,e^	270,93 ± 11^d^	0/4 (0)^b,d,e^	0/4 (0)^b,d,e^	1/4 (25)^b^
	Placebo	2/4 (50)^a,b,c,e^	985,40 ± 64^a,b,e^	725,32 ± 117^ª,b,c,e^	2/4 (50)^a,b,c^	2/4 (50)^a,b,c^	1/4 (25)^b^
	Sulfasalazine	1/4 (25)^a,b,d^	747,49 ± 135^c,d^	327,00 ± 123^d^	2/4 (50)^a,b,c^	2/4 (50) ^a,b,c^	1/4 (25)^b^
15 days	Control	4/5 (80)^b,d,e^	1181,486 ± 201	342,6 ± 60	0/5 (0)^b,c,d^	0/5 (0)^b,c,d^	1/5 (2^b,c^
	Colitis	4/4 (100)^a,c,d,e^	1217,753 ± 218	338,35 ± 28	1/4 (25)^a,d,e^	1/4 (25)^a,e^	3/4 (75)^a,c,d,e^
	Probiotic	4/5 (80)^b,d,e^	1026,708 ± 268	299,18 ± 59	1/5 (20)^a,d,e^	1/5 (20)^a,e^	2/5 (40)^a,b,d,e^
	Placebo	2/4 (50)^a,b,c^	1255,71 ± 153	347,91 ± 2,6	2/4 (50)^a,b,c,e^	1/4 (25)^a,e^	1/4 (25)^b,c^
	Sulfasalazine	3/5 (60)^a,b,c^	961,5913 ± 191	281,41 ± 38	0/5 (0)^b,c,d^	0/5 (0)^b,c,d^	1/5 (20)^b,c^

The small intestine, large intestine and spleen were stained with Hematoxylin–eosin (HE) for analysis under light microscopy and the digital images were analyzed using appropriate software (Image J® 1.50b, National Institutes of Health, USA) by one blind trained researcher^19^. The experimental groups were divided into two different periods for treatment, with animals treated for 5 (n = 4) and 15 days (n = 5) with probiotic (orange juice containing *Pediococcus acidilactici* CE51), placebo (orange juice) or sulfasalazine (10 mg/kg/weight) after DSS administration (3%) to induce colitis. The control group was not subjected to the colitis protocol and treatment.

The following parameters were also specifically analyzed to determine the level of inflammation resulting from inflammation caused by DSS, according to Pegoraro et al.^18^: Intestine (small and large): Interstitial inflammatory infiltrate (0 = absent, 1 = mild, 2 = moderate, 3 = intense); epithelial ulceration (0 = absent; 1 = present and small; 2 = present and extensive); Spleen: white pulp hyperplasia (0 = absent; 1 = present) and red pulp hyperplasia (0 = absent; 1 = present).

^a^Statistically significant difference in relation control group (p < 0.05).

^b^Statistically significant difference in relation colitis group (p < 0.05).

^c^Statistically significant difference in relation probiotic group (p < 0.05).

^d^Statistically significant difference in relation placebo group (p < 0.05).

^e^Statistically significant difference in relation sulfasalazine group (p < 0.05).

The analysis of the small intestine evaluated parameters such as intensity of inflammation and villus measurements. With 5 days of treatment, a lower intensity of inflammation was observed in the probiotic and sulfasalazine group compared to the colitis group (p < 0.05). In the 15-day treatment, a 40% reduction in inflammation was observed in the sulfasalazine group and 20% in the probiotic group (p < 0.05). The measurements of intestinal villi showed a significant increase in the probiotic and placebo group after treatment for 5 days (p < 0.05). However, after 15 days of treatment, no significant changes were observed among the groups.

In the large intestine, the measurement of crypts, intensity of inflammation and presence of ulceration were evaluated. Crypt measurements were higher in the placebo group treated for 5 days (p < 0.05) and did not show significant differences with 15 days of treatment. There was a significant reduction of 25% in the intensity of inflammation and 25% of the presence of ulceration in the probiotic group compared to the colitis group after 5 days, however, with 15 days of treatment a more pronounced reduction was observed in the sulfasalazine group (p < 0.05).

White pulp hyperplasia was evaluated in the spleen. There was a 50% reduction in hyperplasia in the probiotic, placebo and sulfasalazine group after 5 days of treatment (p < 0.05). However, the reduction was 35, 50 and 55% respectively in the probiotic, placebo and sulfasalazine groups with treatment for 15 days compared to the untreated colitis group (p < 0.05).

### Reduction of C-reactive protein levels after 15 days of treatment with *P. acidilactici* CE51 on DSS-induced colitis

**Figure 4:**
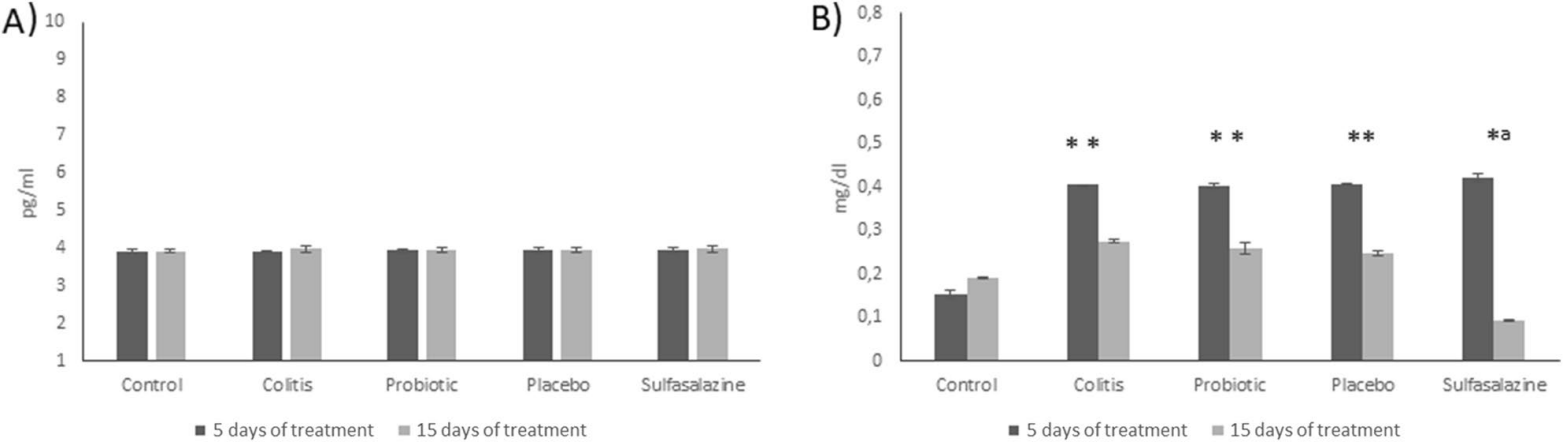
Reduction of C-reactive protein levels after 15 days of treatment with *P. acidilactici* CE51 on DSS-induced colitis. Serum concentrations of TNF-α (**A**) and C-reactive protein (**B**) from mice treated for 5 days (n = 4) and 15 days (n = 5) with probiotic (orange juice containing *Pediococcus acidilactici* CE51), placebo (orange juice) and sulfasalazine (10 mg/kg/weight) after DSS administration (3%) to induce colitis. The concentration of TNF-α and C-reactive protein (CRP) were determined by the enzyme-linked immunosorbent assay (ELISA) and, in the final of the test, the resulting absorbance was measured spectrophotometrically at 450 nm. The control group was not submitted to the colitis protocol. *Statistical difference in relation to the control group (p < 0.05). ^a^Statistical difference in relation to the colitis, placebo and probiotic group (p < 0.05).

The measurement of TNF-α demonstrated similarity between the groups during 5 and 15 days of treatment with probiotic, placebo or sulfasalazine (Fig. 4A). The treatment time impacted the levels of C-reactive protein, with a higher dosage being observed with a 5-day treatment for all groups that had colitis induction (p < 0.05). The sulfasalazine group with 15 days of treatment showed a significant reduction when compared with the other groups (p < 0.05) (Fig. 4B).

### Probiotic orange juice positively modulates the gut microbial communities in DSS-induced colitis

**Figure 5:**
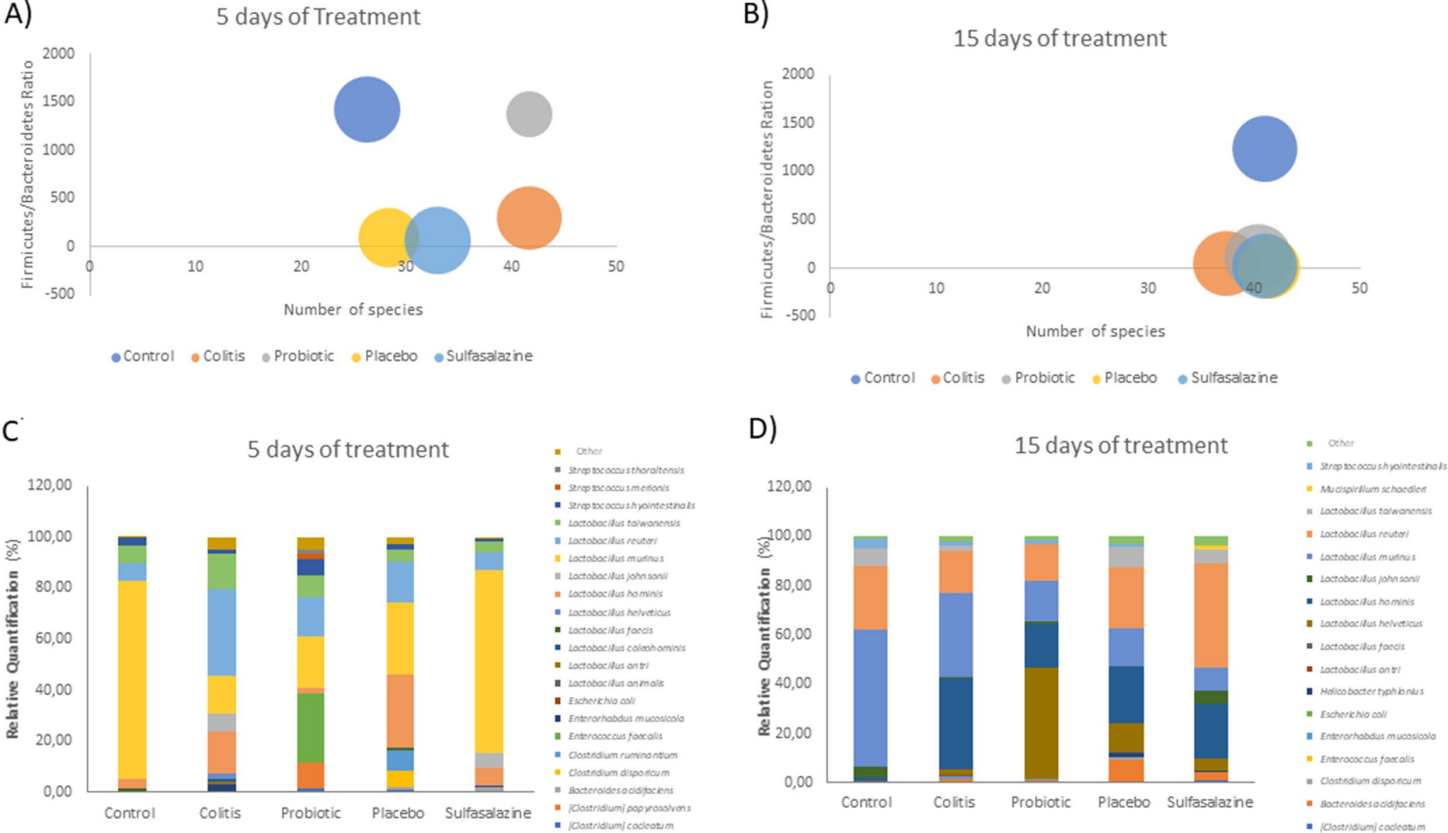
Probiotic orange juice positively modulates the gut microbial communities in DSS-induced colitis. Evaluation of the quantitative ratio of the phylum *Firmicutes/Bacteroidetes* (Y axis), number of species (x axis) and number of specimens of the *Lactobacillaceae* family (circle size) (**A** and **B**). Relative quantity of the most prevalent bacterial species (**C** and **D**). Results obtained from the metagenomic analysis of the pool of feces obtained from mice treated for 5 days (n = 4) and 15 days (n = 5) with probiotic (orange juice containing *Pediococcus acidilactici* CE51), placebo (juice orange) and sulfasalazine (10 mg/kg/weight) after DSS administration (3%) to induce colitis. The control group was not submitted to the colitis protocol.

Figure 5 shows the relationship between species diversity, the phylum *Firmicutes/Bacteroidetes* (*F/B*) ratio and the number of specimens in the *Lactobacillaceae* family. There was a significant increase in the diversity of bacterial species after 5 days of treatment for the probiotic group and colitis group (p < 0.05) (Fig. 5A). There were no significant changes after 15 days of treatment (Fig. 5B).

The quantitative ratio of the phylum *Firmicutes/Bacteroidetes* was reduced in the colitis, placebo and sulfasalazine group after 5 days of treatment (p < 0.05). After 15 days of treatment, a significant decrease was observed in all groups (p < 0.05). The number of specimens of the *Lactobacillaceae* family, represented by the size of the circle, was reduced in about 50% for the probiotic group after 5 days of treatment (p < 0.05). However, such a reduction was not observed after 15 days of treatment.

The relative quantity of bacterial species after 5 days of treatment had a predominance of *Lactobacillus murinus* in the control group and sulfasalazine with approximately 70% each (Fig. 5C). Colitis, probiotic and placebo groups had a significant reduction in *Lactobacillus murinus* and an increase in the proportion of *Lactobacillus hominis* and *Lactobacillus reuteri*. However, the probiotic group also showed an increase in *Enterococcus faecalis* (p < 0.05).

After 15 days of treatment, a reduction in the relative amount of *Lactobacillus murinus* was observed in the probiotic group compared to the control group (p < 0.05) (Fig. 5D). On the other hand, there was an increase in *Lactobacillus hominis* for the colitis, probiotic, placebo and sulfasalazine group (p < 0.05). In the probiotic group, a significant increase in *Lactobacillus helveticus* was also observed (p < 0.05).

## Discussion

In view of the need for studies on compounds that act in the treatment and prevention of ulcerative colitis, this study evaluated and found the positive effect of the probiotic drink containing *P. acidilactici* CE51 regarding the reduction of inflammatory parameters presented in histopathological analyzes and modulation of the gut microbiota composition.

Adequate concentration and administration of probiotic strains is essential to induce their positive effect, requiring an inoculum of at least 10^6^ CFU/mL of the live probiotic strain at the time of consumption^29^. In the present study, a concentration of 10^8^ CFU was used/mL and the effectiveness of this administration in reducing the effects of induced colitis has been demonstrated. Similar results were obtained by Srutkova et al.^30^ and Mansour et al.^14^, respectively for the use of 2 × 10^8^ CFUs of *B.* *longum* ssp. *Longum* CCM 7952 (Bl 7952) and CCDM 372 (Bl 372) in the prevention of DSS-induced colitis, and orally daily dose containing CFU 10^9^ *Pediococcus acidilactici* strains induction for colitis by acetic acid.

Histopathological analyzes showed that the group that received *P. acidilactici* CE51 for 5 days showed a decrease in the intensity of inflammation in the intestines, an increase in intestinal villi, absence of ulceration and a reduction in white pulp hyperplasia when compared to the untreated colitis group. After 15 days of treatment with the *P. acidilactici* CE51, the evidence was less pronounced.

The inflammatory process in the intestinal mucosa results in reduced villi, digestive, and absorptive activities. The probiotic strains compete with pathogenic microorganisms for binding sites, reducing the occurrence of diarrhea and favoring the absorption of nutrients, this mechanism directly influences the recovery of the intestinal mucosa, increasing the height of the villi^31^. This positive effect could be observed in the probiotic group treated for 5 days.

The white pulp present in the spleen is particularly lymphatic and plays a fundamental role in the immunological activity^32^, which justifies the hyperplasia of the white pulp, since this increase is directly related to the proliferation of leukocytes due to a severe inflammatory process induced in these animals.

Evidence suggests that UC leads to complications in gastric mucosal lesions, involving tissue inflammation and destruction, potentially causing gastritis and ulcers^33^. Kwon et al.^34^ show that DSS-induced colitis is associated with adipose tissue dysfunction and disrupted hepatic lipid metabolism leading to hepatosteatosis and dyslipidemia in mice. However, in the present study there were no histopathological differences in both organs and tissues^35^.

Orange juice, alone, also had the potential to reduce inflammatory parameters in murine-induced colitis. There was a reduction in the intensity of inflammation in the small intestine, an increase in the height of the villi and crypts and a reduction in spleen hyperplasia. Citrus juice, especially orange juice, is associated with improved immunity, as it is a source of vitamin C, flavonoids and bioactive compounds, which together have a potential anti-inflammatory, antioxidant effect and improved endothelial function^36^. This fact highlights the importance of orange as a food matrix that can reduce inflammatory biomarkers and serve as an attractive strategy to counteract these types of stresses.

Although no evidence has been found in the literature using *P. acidilactici* CE51 in the treatment of ulcerative colitis, the results of improvement in the histological parameters, as well as aid in the protection of the epithelial barrier is in agreement to the findings of Duary et al.^37^. In that work, the anti-inflammatory and immunomodulatory efficacy of probiotic strains of *Lactobacillus plantarum* Lp91 was observed as a prevention of ulcerative colitis when compared to the untreated colitis group and the colitis group treated with *L. plantarum* CSCC5276. The animals received the live cells at 10^9^ CFU resuspended in 25 μL in PBS by mouth^37^.

Wang et al.^38^ verified the mixture (1:1, 10^9^ CFU/mL) of *Lactobacillus plantarum* ZDY2013 and *Bifidobacterium bifidum* WBIN03 as an excellent strategy of antioxidant activity and immune stress relief in mice with DSS-induced colitis. Those animals were treated for 7 days with probiotics, with a reduction in pro-inflammatory cytokines, such as TNF-α, and an increase in antioxidant factors, such as SOD1, SOD2, GPX2. Groeger et al.^39^ when using 1 × 10^10^ CFU viable *Bifidobacterium infantis* 35624 in the treatment of gastrointestinal and non-gastrointestinal inflammatory disorders, concluded that this strain reduced the levels of C-reactive protein and TNF-α, but had no impact on ulcerative colitis.

The fact that the measurement of TNF-α did not give statistical difference between the groups treated for 5 and 15 days in the present study, corroborates with a systematic review where it was found that the use of probiotics does not impact the measurements of serum IL10 and TNF-α^40^. Regarding the C-reactive protein dosage, a significant reduction was demonstrated only in the sulfasalazine group after 15 days of treatment. The dosages of C-reactive protein and TNF-α were not influenced by the administration of *P. acidilactici* CE51 probable because they are considered markers of systemic inflammation and due to the short term of administration of the CE51 probiotic^41^. Studies evaluating its application for a longer period of time or its concomitant use with sulfasalazine are necessary to further evaluate its potential to improve the imumodulatory response of colitis induced in animals.

Studies have shown that during an IBD there is a significant reduction in potentially beneficial species such as *Bifidobacterium, Lactobacilli*, and *Firmicutes* and *Bacteroidetes* phyla, which make up the healthy intestinal microbiota. On the other hand, there may be a large increase in pathogenic strains under IBD conditions such as: *Campylobacter jejuni, Salmonella enterica, Vibrio cholera* and *Escherichia coli* (*E. coli*) and *Bacteroides fragilis*^42^.

The gut microbiota is characterized as a diverse microbial community present in the gastrointestinal tract that maintains gut symbiosis in the host. In it, the two dominant bacterial phyla, *Firmicutes* and *Bacteroidetes*, are present, which consist of more than 90% of the total community. The relationship between the two is expressed as the *Firmicutes/Bacteroidetes* ratio, commonly related to intestinal pathological conditions^43,44^. As the phyla *Firmicutes* and *Bacteroidetes* predominate in healthy intestines and help produce epithelial metabolites. In IBD, the microbiota is represented with relative absence of these phyla and with exacerbated presentation of *Enterobacteriaceae* and *Fusobacteria*^45^.

In intestinal biopsies and stool samples from UC, it is common to observe disproportions in the *F/B* ratio, in which *Firmicutes* are decreasing and *Bacteroidetes* are generally abundant^46^. In the present study, a significant decrease in *F/B* in the colitis, placebo and sulfasalazine groups treated for 5 days and maintaining the proportion in the probiotic group equivalent to the control group. This data highlights the maintenance of a healthy intestinal microbiota in the group that received *P. acidilactici* CE51, since the decrease in the abundance of *Firmicutes* and *Bacteroidetes* is related to the development and advancement of the disease, resulting from bacterial dysbiosis in inflamed sites compared to non-inflamed tissues^47^.

Strains of the *Lactobacillaceae* and *Bifidobacteriaceae* families are characterized as the most common probiotics. The family *Lactobacillaceae* (phylum *Firmicutes*) is predominant in the gut microbiota, comprising 31 genera, principally *Lactobacillus* species. The presence of *Lactobacillaceae* in the gut is important for preventing colonization by enteropathogenic bacteria through competitive adhesion and aggregation to binding sites. In addition, they demonstrate a protective effect on the intestinal tract, as they increase immunity and produce antimicrobial substances that inhibit pathogens, thus exerting antagonism against them^48,49^.

In the intestinal lumen, microbial genera predominate that can be identified in the feces, such as *Bacteroides*, *Bifidobacterium*, *Streptococcus*, *Enterobacteriacae*, *Enterococcus*, *Clostridium*, *Lactobacillus* and *Ruminococcus*^50^. The number of specimens from the *Lactobacillaceae* family showed a significant reduction after the 5-day period, possibly attributed to an increase in the species *Enterococcus faecalis* belonging to the *Enterococcaceae* family. Probably, this fact may be related to the condition of competition of different bacterial species for nutrients and adhesion to the mucosa in this condition^51^.

The results presented here did not show the presence of *P. acidilactici* CE51 in fecal samples, and this may be related to the application of the metagenomic technique, which has sensitivity limitations when it comes to a complex sample and which presents a microbiota with a high diversity of species with possible inhibitors; as is the case with stool analysis^52^. In addition, the bacteria may have been unable to colonize and compete with the murine gastrointestinal microbiota. Similar results were observed in the study by Grimm et al.^53^, where there was treatment for *B. bifidum* S17/pMGC (2 × 10^9^ CFU in 20 μL of PBS), however, fecal levels were decreased during the pretreatment and DSS phase, making it difficult to detect them in the feces.

It was possible to observe a positive modulation of the species that make up the intestinal microbiota with the treatment of the *P. acidilactici* CE51. It is known that *Lactobacillus murinus* is the most prevalent and prevalent isolate found in this rodent species chosen for the experiment, as demonstrated in the control and sulfasalazine group with 5 days of treatment^54^. When analyzing the animals treated for 15 days, it was noted that all groups of animals showed an increase in *Lactobacillus hominis*, one of the most common species found in the intestine^55^. In addition, the group that received *P. acidilactici* CE51 also had a significant and predominant increase in *Lactobacillus helveticus*. These microorganisms assist in intestinal positive modulation, can improve the inflammatory response and are considered effective in the treatment of diarrhea associated with antibiotics and liver damage^56^.

In the literature, the use of anti-inflammatory drugs and probiotic mixtures is highlighted as the main means of combating UC. Palumbo et al.^57^ showed that patients treated with mesalazine and a mixture of probiotics containing *Lactobacillus salivarius*, *Lactobacillus acidophilus* and *Bifidobacterium bifidus* strain BGN4 (Acronelle®, Bromatech srl, Milan, Italy) showed significant improvement in relation to those treated with mesalazine alone. This demonstrates that the combination of therapies generates positive effects on the patient's health and the probiotic mixtures help in the effective improvement of the clinical condition, which can potentiate anti-inflammatory therapy. A study emphasized the potential of capsules containing 2.5–25 × 10^9^ viable bacteria (Mutaflor 100 mg; Ardeypharm GmbH, Herdecke, Germany)of *Escherichia coli Nissle 1917* (EcN) probiotic strain to maintain remission in patients with ulcerative colitis and be equivalent to the standard treatment with mesalazine^58^. Other researchers, applying the same strain in experiments with mice, reported the benefits in relation to pathological and/or physiological parameters, and daily 10^8^ CFU of live bacteria and 1.5–2 × 10^8^ CFU (Mutaflor mite, Ardeypharm, Germany) EcN twice daily by oral were applied, respectively^59,60^.

As a result of these data, a comparison can be established with the findings of the present study, since *P. acidilactici* CE51 was shown to be more effective than the treatment with the sulfasalazine drug in some parameters such as increased villi in the small intestine, reduced ulceration and intensity of inflammation in the large intestine and maintenance of the proportion of the phylum *F/B* when used for 5 days in the treatment.

Thus, these data suggest that the benefits obtained by the probiotic drink containing *P. acidilactici* CE51 are more evident in 5 days of treatment. After this period, the organism is probably able to obtain an inflammatory response for the recovery of tissue damage generated by the induction of colitis. Furthermore, it is worth mentioning that the intestine has an accelerated cell multiplication and renewal process, which may also favor its resourcefulness in recovery^61^.

## Conclusion

In conclusion, the results showed the potential beneficial effect of the application of probiotic orange juice containing *Pediococcus acidilactici* CE51 as a promising therapy in the treatment and management of colitis in murine, being able to maintain the integrity of the intestinal barrier, maintain body weight, assist in the decline of DAI and positively modulated the gut microbiota.

## Data Availability

Derived data supporting the findings of this study are available from the corresponding author on request.
